# Efficacy of percutaneous cement discoplasty combined with PVP for the treatment of stage III Kümmell disease with an adjacent disc vacuum sign

**DOI:** 10.3389/fsurg.2025.1538964

**Published:** 2025-02-18

**Authors:** Liehua Liu, Pei Li, Lei Luo, Chen Zhao, Huilin Zhang, Deqiang Liu, Qiang Zhou

**Affiliations:** Bone and Trauma Center, Third Affiliated Hospital of Chongqing Medical University, Chongqing, China

**Keywords:** percutaneous cement discoplasty, Kümmell disease, percutaneous vertebroplasty, internal fixation and fusion, disc vacuum sign

## Abstract

**Objective:**

To observe the efficacy of percutaneous cement discoplasty (PCD) combined with PVP for the treatment of stage III Kümmell disease (KD).

**Methods:**

A total of 33 patients with stage III KD who underwent surgical treatment were divided into the PCD + PVP (PP) group (*n* = 20) and the internal fixation and fusion (IFF) group (*n* = 13). The observation indices included demographic characteristics, perioperative information, and clinical and imaging indicators, including the visual analog score (VAS), Oswestry disability index (ODI), Cobb angle, and height ratio of the injured vertebra and its adjacent intervertebral disc (H_v__+__d_, RH_v__+__d_).

**Results:**

The operation time, blood loss, duration of bedrest, length of stay and cost of hospitalization were significantly lower in the PP group than in the IFF group (*P* < 0.001). There were no significant differences in the VAS score or ODI between the two groups before the operation, after the operation or at the last follow-up (*P* > 0.05). The RH_v_ _+_ _d_ in the IFF group was significantly higher than that in the PP group after surgery and at the last follow-up (*P* < 0.05). The Cobb angle in the IFF group was significantly smaller after the operation than before the operation (*P* = 0.007). The incidence of complications in the PP group was lower than that in the IFF group (*P* = 0.018).

**Conclusions:**

PCD combined with PVP for the treatment of stage III KD with an adjacent disc vacuum sign is effective and may be the next best thing to IFF, especially for elderly patients with complex underlying diseases and great surgical risks.

## Introduction

Kümmell's disease (KD) is a specific type of osteoporotic vertebral compression fracture (OVCF) (1, 2). KD, also known as posttraumatic vertebral osteonecrosis, is caused by posttraumatic vertebral ischemia and vascular necrosis, resulting in vertebral nonunion, vertebral vacuum, or vertebral pseudoarticulation (3). The main clinical features of KD are intractable low back pain and kyphosis, and some patients have neurological symptoms (4). Delayed vertebral collapse and characteristic intramural vacuum can be found on imaging (5).

Conservative treatment of KD has a poor effect, and most doctors advocate surgical treatment (6, 7). Stage III KD is often characterized by an incomplete posterior wall of the vertebral body and may be associated with spinal stenosis, kyphosis or even nerve injury (8). Most scholars recommend internal fixation and fusion surgery (9, 10). For elderly patients with complex underlying diseases and poor tolerance, the risk of internal fixation and anesthesia is high (11). In 2015, Vargar et al. (12) introduced a minimally invasive surgery called percutaneous cement discoplasty (PCD), which involves the injection of bone cement into the degenerated intervertebral disc to achieve intervertebral stabilization and indirect nerve decompression. Recent meta-analyses and biomechanical research have confirmed that PCD is an alternative surgery for treating degenerative lumbar diseases in elderly patients who cannot undergo open surgery (13, 14).

The authors performed PCD and percutaneous vertebroplasty (PVP) in elderly patients with stage III KD without neurological symptoms and reported good short-term efficacy.

## Materials and methods

A total of 33 patients with stage III KD who underwent surgical treatment between January 2019 and September 2023 were retrospectively studied. The patients were divided into the PCD + PVP (PP) group (*n* = 20) and the internal fixation and fusion (IFF) group (*n* = 13). This study was approved by the Ethics Committee of the XX Medical University (2025–15). Each participant provided written consent before starting the operation.

The inclusion criteria were as follows: (1) aged above 60 years; (2) recurrent chronic low back pain for ≥3 weeks; (3) MRI showing low vertebral signal intensity in T1 images and high signal intensity in fat inhibition images and KD diagnosis; (4) vacuum phenomenon in the adjacent intervertebral disc on CT; (5) vertebral instability, kyphotic deformity, posterior wall fracture of the vertebral body and spinal canal occupation, or upper or lower endplate fracture; and (6) more than 6 months of follow-up.

The exclusion criteria were as follows: (1) pathological vertebral fractures due to vertebral tumors, spinal tuberculosis, or spinal infections; (2) neurological symptoms from nerve root or spinal cord compression; (3) MRI showing that the bone fragment had compressed the spinal cord and caused local depression; (4) previous history of spinal fusion; and (5) history of abnormal bleeding and coagulation dysfunction.

After the patient was admitted, the stage III KD and its treatment methods (conservative or surgical) were described in detail. In terms of the choice of surgical method, the patient was informed that internal fixation and fusion were recommended first, and PCD combined with PVP surgery was the secondary surgical option. Patients were informed of the advantages and disadvantages of the two surgical methods in detail. In particular, PCD combined with PVP, which may be a temporary treatment that provides limited spinal stability, may be followed by secondary exacerbation of kyphosis, spinal canal occupation and compression of the spinal cord or nerves and may require further spinal canal decompression or deformity correction surgery. The chief surgeons in the study were the same people, and the surgical assistants were all members of the team.

## Surgical methods

**Figure 1:**
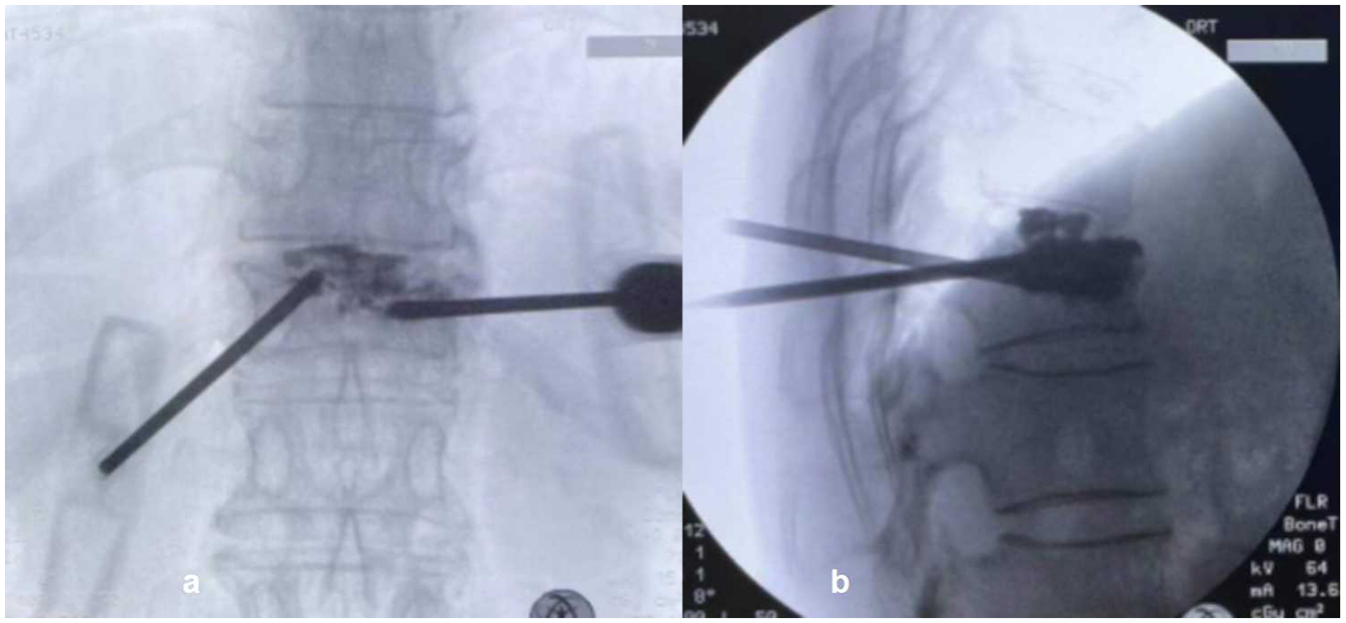
The puncture method in PP group. **(a)** The working channel of the injured vertebra was punctured through the pedicle on the left side; **(b)** the intervertebral space was punctured through the pedicle on the right side to pierce the endplate. In operative x-ray, the bone cement in the vertebral body was diffused through the endplate and was connected to the bone cement in the intervertebral disc.

Group PP. Patients were positioned prone, with a pillow under the chest and iliac ridge. The spinous process of the injured vertebra was centered via x-ray fluoroscopy, and the resulting pedicle projection was symmetric. Generally, local anesthesia was selected. The working channel of the injured vertebra was punctured through the pedicle on one side, and the intervertebral space was punctured through the pedicle on the other side to pierce the endplate (Figure 1), or over the transverse process, outside the superior articular process, close to the outer part of the pedicle and through the Kabin triangle. Bone cement was injected under strict fluoroscopy. If the injection pressure was too high or the cement was tended to diffuse into the spinal canal, bone cement injection should be stopped. Preoperative CT revealed a vacuum in the vertebral space connected to the fractured endplate in some patients. The direction and angle of the working channel used for puncture into the vertebral body were adjusted during the operation, and bone cement was injected into the vacuum of the vertebral space through the broken endplate.

Group IFF. Under general anesthesia, the patient was positioned prone, with the chest and iliac ridge raised, and a posterior median incision was made to expose the bilateral laminae and the entry point of the pedicle screw. Pedicle screws were inserted into the injured vertebra as far as possible, and long segment fixation or short segment fixation was selected. Long segment fixation refers to the placement of pedicle screws in at least two normal vertebrae above or below the injured vertebra, and short segment fixation refers to the placement of pedicle screws in at least one normal vertebra above or below the injured vertebra. A titanium rode with an appropriate curvature is used to restore the height of the injured vertebra and correct the kyphotic deformity. The posterolateral laminae and facet joints were roughened and fused with allogeneic bone. Symptomatic treatment as well as measures to prevent postoperative infection were initiated. A thoracolumbar brace was worn for three months. The internal fixation did not need to be removed after surgery.

Postoperative anti-osteoporosis therapy should be emphasized, and bisphosphonates plus calcium and active vitamin D are the first choice. Bisphosphonates were administered after teriparatide to some with financial means. Patients were followed up at 1, 3, 6, and 12 months after surgery and then once a year. When the follow-up time was up, the nurse would call the patient back to the hospital. In the outpatient department, the attending doctor was the chief surgeon. He inquired about the patient's symptoms, performed VAS and ODI scores, ordered relevant imaging examinations, and measured the Cobb angle, the heights of vertebral body and disc, observed whether internal fixation failed or screws loosen, etc.

## Observation index

The demographic data, including patient age, sex, bone mineral density (T score), and follow-up time, were collected. Perioperative information: operation time, blood loss, amount of bone cement, time to ambulate from bed, length of hospital stay and cost of hospitalization.

Clinical and imaging indicators. The visual analog scale (VAS) score, Oswestry disability index (ODI), Cobb angle, and ratio of the height of the injured vertebra to that of the adjacent intervertebral disc (H_v__+__d_, RH_v__+__d_) were recorded before surgery, after surgery (PP group: 1–2 days after surgery; IFF group: 1 month after surgery), and at the last follow-up.

RH_v__+__d_ = (anterior vertebral height + anterior height of intervertebral disc)/(posterior vertebral height + posterior height of intervertebral disc). In the PP group, the extension of the intervertebral space may be due to either the inclusion of the upper or lower intervertebral space or the two adjacent intervertebral spaces (PCD_up_ + PVP, PVP + PCD_down_, PCD_up_ + PVP + PCD_down_). In the IFF group, if the injured vertebra was implanted with pedicle screws, the H_v__+__d_ refers to the height of the injured vertebra plus the upper intervertebral space; if the injured vertebra was not implanted with pedicle screws, the H_v__+__d_ refers to the height of the injured vertebra plus the upper and lower intervertebral space. The Cobb angle was formed by the upper endplate of the upper normal vertebra and the lower endplate of the lower vertebra.

Intraoperative and postoperative complications included bone cement leakage, bone cement allergy, massive bleeding of more than 800 ml, lung infection, deep vein thrombosis of the lower extremity, incision infection, internal fixation loosening, proximal junctional failure (PJF), etc.

## Statistical analysis

SPSS 23.0 statistical software (SPSS, Inc., Chicago, IL, USA) was used for analysis. Quantitative data are expressed as the mean ± standard deviation. An independent two-sample *t*-test was used to identify significant differences between the two groups. Counting data were tested by using the the chi-square test. A *P* value < 0.05 was considered to indicate statistical significance.

## Results

1. The demographic data and perioperative information of the patients in the two groups are shown in Table 1.

There were no significant differences in sex, age, bone mineral density or follow-up time between the two groups. Two patients in the PP group underwent general anesthesia induction, while all patients in the IFF group underwent general anesthesia induction. Fifteen PCD plus PVP (PCD_up_ + PVP) procedures were performed in the upper disc, 4 PCD plus PVP procedures were performed in the upper and lower discs (PCD_up_ + PVP + PCD_down_), and 1 PCD plus PVP procedure was performed in the lower disc (PVP + PCD_down_). In the IFF group, 9 patients underwent long segment fixation, and 4 patients underwent short segment fixation. The operation time, blood loss, duration of bedrest, length of stay and cost of hospitalization in the PP group were significantly lower than those in the IFF group (*P* ≤ 0.001).

2. Clinical and imaging indicators in the two groups are shown in Table 2.

**Table 1:** The demographic database, perioperative information in the two groups.

	*n* (Group PP) = 20	*n* (Group IFF) = 13	*t*/2	*P*
Sex (male: femal)	4:16	3:10	0.045^t^	0.833
Age (years)	71.10 ± 6.79	65.46 ± 8.99	2.051^2^	0.051
Bone mineral density	−2.93 ± 0.96	−2.40 ± 1.18	−1.403^t^	0.171
General anesthesia: local anesthesia	2:18	13:0	25.740^2^	0.000
Operation time (min)	40.00 ± 14.14	126.15 ± 47.18	−6.400^t^	0.000
Blood loss (ml)	2.90 ± 0.97	226.92 ± 192.15	−4.204^t^	0.001
Amount of bone cement (vertebra) (ml)	2.30 ± 0.50			
Amount of bone cement (disc) (ml)	1.63 ± 0.52			
PCD_up_ + PVP	15			
PVP + PCD_down_	1			
PCD_up_ + PVP + PCD_down_	4			
Long segment fixation: short segment fixation		9:4		
Time to ambulate from bed (day)	1.90 ± 0.55	5.23 ± 1.30	−8.734^t^	0.000
Length of hospital stay (day)	18.46 ± 6.53	5.20 ± 2.28	−8.389^t^	0.000
Cost of hospitalization (RMB)	18294.21 ± 7178.05	71550.31 ± 18797.10	−9.763^t^	0.000
Follow-up time (mon)	20.10 ± 12.79	23.30 ± 10.44	−0.754^t^	0.456

t: independent sample *t*-test statistic; 2: chi-square test statistic. The independent sample *t*-tests are all two-tailed tests.

PCDup + PVP, percutaneous cement discoplasty in the upper disc combined with percutaneous vertebroplasty; PVP + PCD_down_, percutaneous cement discoplasty in the down disc combined with percutaneous vertebroplasty; PCD_up_ + PVP + PCD_down_, percutaneous cement discoplasty in the upper and down discs combined with percutaneous vertebroplasty.

**Table 2:** Clinical and imaging indicators in the two groups.

	*n* (Group PP) = 20	*n* (Group IFF) = 13	*t*	*P*
VAS				
pre-operation	6.10 ± 1.17	6.15 ± 1.21	−0.128	0.899
postoperation	2.45 ± 0.94	2.77 ± 0.83	−0.993	0.329
final follow-up	2.05 ± 0.60	1.69 ± 0.85	1.410	0.169
*P*_1_	0.000	0.000		
*P*_2_	0.120	0.003		
ODI				
pre-operation	64.80 ± 10.93	63.38 ± 11.08	0.361	0.720
postoperation	25.95 ± 8.74	29.69 ± 8.40	−1.220	0.232
final follow-up	14.90 ± 4.06	15.69 ± 6.30	−0.440	0.663
*P*_1_	0.000	0.000		
*P*_2_	0.000	0.000		
RH_v_ _+_ _d_				
pre-operation	59.55 ± 18.56	61.92 ± 15.66	−0.381	0.706
postoperation	73.85 ± 18.04	87.11 ± 9.93	−2.415	0.022
final follow-up	67.50 ± 15.87	82.92 ± 9.05	−3.173	0.003
Loss at final follow-up	6.39 ± 4.80	4.09 ± 4.71	1.350	0.187
*P*_1_	0.018	0.000		
*P*_2_	0.000	0.282		
Cobb				
pre-operation (X)	22.04 ± 15.76	26.99 ± 15.84	−0.879	0.386
pre-operation (CT)	15.23 ± 12.91	20.65 ± 12.03	0.210	0.235
postoperation (X)	15.89 ± 14.46	12.05 ± 9.23	0.848	0.403
final follow-up (X)	18.36 ± 14.62	14.25 ± 10.38	0.878	0.387
Loss at final follow-up	2.48 ± 1.51	2.20 ± 1.29	0.540	0.593
*P*_1_	0.206	0.007		
*P*_2_	0.593	0.573		

*P*_1_, pre-operation vs. post-operation; *P*_2_, final follow-up vs. post-operation.

There were no significant differences in the VAS score or ODI between the two groups before the operation, after the operation or at the last follow-up (*P* > 0.05). The postoperative VAS and ODI scores of the two groups were significantly better than those before the operation (*P* = 0.000). The preoperative RH_v__+__d_ was similar between the two groups, but the RH_v__+__d_ after surgery and at the last follow-up was significantly greater in the IFF group than in the PP group (*P* < 0.05). There was no significant difference in RH_v__+__d_ loss between the two groups.

There were no significant differences in the Cobb angle before, after, or at the last follow-up between the two groups. The postoperative Cobb angle in the PP group was smaller than that before the operation, but the difference was not significant (*P* = 0.206). However, the Cobb angle in the IFF group was significantly smaller after the operation than before the operation (*P* = 0.007). The Cobb angle on preoperative CT was smaller than that on preoperative x-ray in both groups, but there was no significant difference (*P* > 0.05). In the PP group, the postoperative Cobb angle on x-ray was similar to the preoperative Cobb angle on CT, which was approximately 15°.

3. The incidence of complications in the PP group was significantly lower than that in the IFF group (*P* = 0.018) (Table 3). In the PP group, there was 1 case of bone cement leakage and 1 case of adjacent vertebral fracture. In the IFF group, there were 2 cases of pulmonary infection, 1 case of incision infection, 1 case of deep vein thrombosis, 1 case of internal fixation loosening, and 1 case of proximal junctional failure. None of the patients required revision surgery during the follow-up period.

**Table 3:** Intraoperative and postoperative complications in the two groups.

Complication	*n* (Group PP) = 20	*n* (Group IFF) = 13	X2	*P*
Bone cement leakage	1			
Lung infection		2		
Incision infection		1		
Deep vein thrombosis of lower extremity		1	5.607	0.018
Adjacent vertebral fracture	1			
Internal fixation loosening		1		
Proximal junctional failure		1		

**Figure 2:**
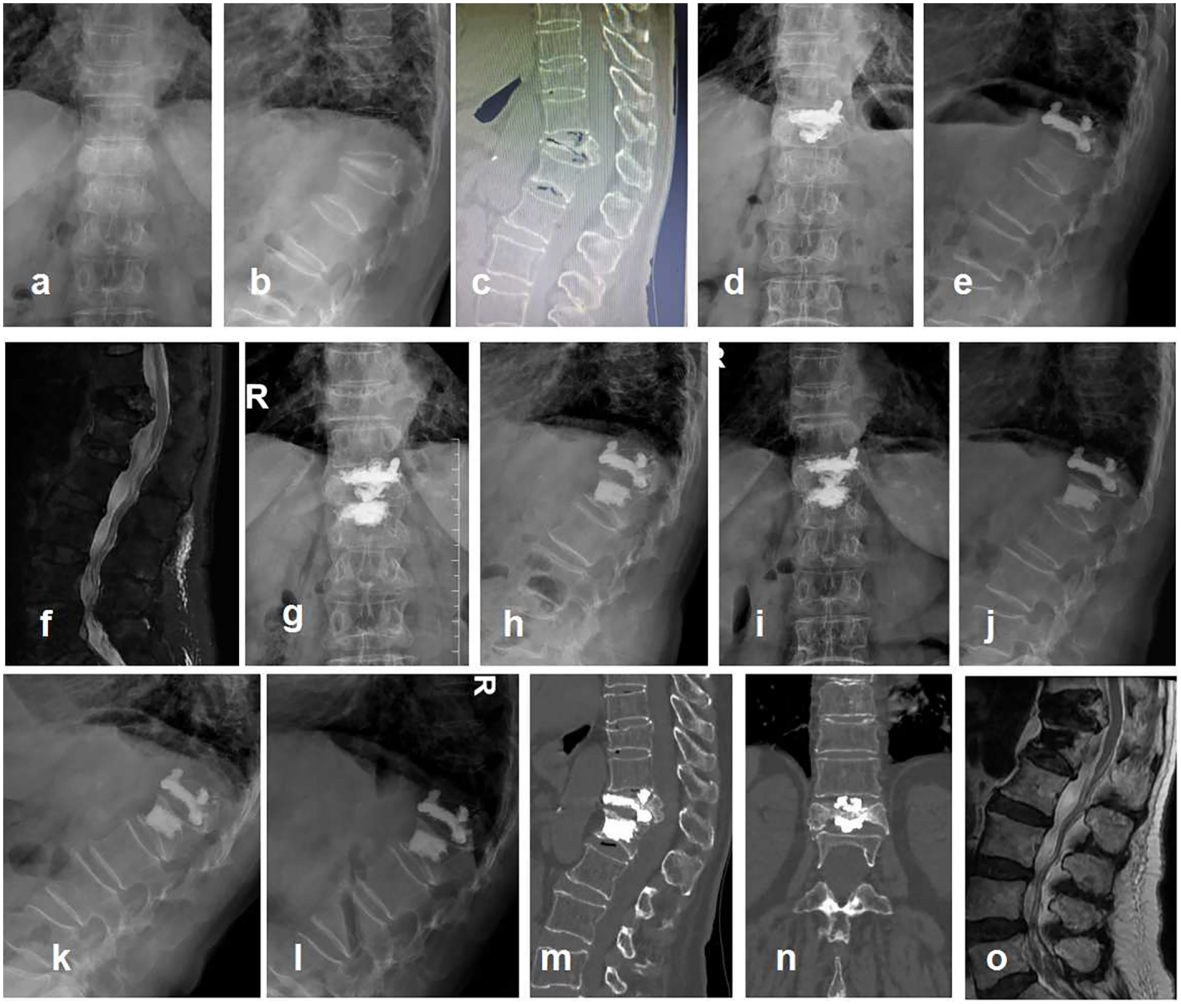
Typical case, a 79-year-old female presented with low back pain for half a year. She had been hospitalized in many hospitals and was afraid of open surgery. Diagnosis: T12 KD, secondary thoracolumbar kyphosis, severe osteoporosis. Surgical methods: T12 PVP + T11/T12 PCD + T12/L1 PCD. VAS was 6, 3, and 1 before surgery, after surgery, and 12 months after surgery, respectively. ODI was 63, 31, and 16 before surgery, after surgery, and 12 months after surgery, respectively. Cobb angle was 41.85°, 39.32°, and 40.23° before surgery, after surgery, and 12 months after surgery, respectively. RH_v_ _+_ _d_ was 0.46, 0.51, and 0.50 before surgery, after surgery, and 12 months after surgery, respectively. **(a,b)** Preoperative anteroposterior and lateral x-rays, T12 severe compression fracture with kyphosis; **(c)** preoperative CT, disc vacuum sign in T11/T12 and T12/L1 intervertebral disc; **(d,e)** postoperative anteroposterior and lateral x-rays, the bone cement in vertebral body (T12) was connected to the bone cement in adjacent T11/T12 and T12/L1 intervertebral discs; **(f)** 1 month after surgery, adjacent vertebral fracture (L1); **(g,h)** L1 PVP; **(i–o)** 12 months after surgery. It was not observed that the bone cement injured the endplate, such as the inferior endplate of T11 and the superior endplate of L1. There was no evidence of bone cement displacement. **(i,j)** Anteroposterior and lateral x-rays; **(k,l)** lumbar hyperextension and hyperflexion x-rays; **(m,n)** CT showed no cement displacement or endplate rupture; **(o)** MRI showed no direct compression of the spinal cord.

Typical case (Figure 2).

## Discussion

Some patients with KD have endplate-intervertebral disc complex injury, which are characterized by the worsening of adjacent intervertebral disc degeneration, increased local kyphosis, decreased intervertebral space height and vacuum signs in the intervertebral disc (15). Currently, there is some controversy regarding the treatment of KD. Conservative treatment is minimally effective for pain relief. After PVP, the vertebral body is strengthened, and low back pain is alleviated; however, intervertebral instability still occurs, intervertebral disc degeneration cannot be prevented, and local kyphosis aggravation cannot be stopped (16). Although traditional osteotomy and pedicle screw fixation and fusion surgery for the correction of kyphosis and segmental instability are considered classic surgeries, they are too risky for elderly individuals (17). After preoperative communication, some patients do not agree to undergo open surgery. Whether augmentation of the vertebral body and adjacent intervertebral space can be used as a temporary treatment for some stage III KD or as a bridging therapy before open surgery was the original intention of the authors.

The bone cement in the intervertebral space may have some effect on the intervertebral disc. Zhao et al. (18) showed that acute trauma to the intervertebral disc and leakage of cement into the intervertebral disc are both considered to accelerate intervertebral disc degeneration, resulting in a decrease in the height of the intervertebral space and an increase in the local kyphotic angle. Jamjoom et al. (19) suggested that moderate and high disc cement leakage were associated with a higher disc degeneration score (*P* = 0.04295), but no difference was found between the MRI disc degeneration grade and the incidence of adjacent vertebral fractures. Rose et al. (20) analyzed bone cement leakage during PVP and percutaneous kyphoplasty (PKP). The leakage rates of PVP and PKP were 39.3% (213/532) and 28.9% (143/493), respectively, but no clinical significance was found. Riesner et al. (21) reported that both balloon kyphoplasty and radiofrequency kyphoplasty were associated with high rates of bone cement leakage but were rarely associated with clinically significant complications. In the PP group, there were vacuum signs in the intervertebral disc, and the height of the intervertebral space decreased significantly. The maintenance of intervertebral height after bone cement injection may reduce the compressive stress of the intervertebral disc.

PCD is an emerging technique that was first used by Varga et al. (12) This technique has been used in elderly individuals to treat low back pain associated with degenerative lumbar scoliosis (22, 23), axial pain caused by severe lumbar disc degeneration (24), lumbar instability (25), lumbar disc herniation (26), etc. Biomechanical research confirmed the effectiveness of PCD in restoring axial stiffness of spinal segments (27). Compression tests have shown that PCD can be used as an alternative to fusion surgery in patients with painful disc degeneration with vacuum signs. Li et al. (28) suggested that bone cement and endplate fusion may have more biomechanical advantages, including reduced incidences of implant subsidence and displacement and increased spinal stability. Since PCD can be used to treat lumbar disc degeneration, it could also be used for treating stage III KD patients with adjacent disc degeneration.

The present study revealed that although anterior column height recovery and kyphotic correction were not as good as those in the IFF group, the ODI and VAS score in the PP group were similar to those at approximately 2 years of follow-up. Kyphosis does not necessarily correlate with quality of life (29). The length of stay, medical cost and duration of bedrest in the PP group were significantly greater than those in the IFF group. Notably, the incidence of complications in the IFF group was significantly higher than that in the PP group, especially for one patients with loosening of the internal fixator and for one patients with PJF. The patient with PJF did not undergo reoperation and was treated conservatively instead. In the PP group, the extent of surgical trauma was minimally invasive, equal to or even slightly better than that in the IFF group. Preoperative x-ray images were taken while the patients were in the standing position, and preoperative CT images were taken in the supine position. Therefore, the Cobb angle on preoperative CT was approximately 6–7° smaller than that on x-ray, which reflects the degree of intervertebral instability before surgery.

For stage III KD patients with adjacent disc degeneration, there are several precautions for PCD surgery. First, in the prone position, the anterior height of the intervertebral space increases, and the injection of bone cement into the intervertebral space helps to restore the height of the anterior column of the spine and correct kyphosis. The spinous process of the injured vertebra can be properly pressed before and during the injection of bone cement to facilitate the distribution of bone cement. Second, the intervertebral cement and the vertebral cement are connected as much as possible to enhance the anchoring effect of the bone cement. Bone cement can be injected simultaneously through two channels punctured separately in the vertebral body and intervertebral space, or the vertebral channel can be retreated until the endplate ruptures to complete intervertebral space bone cement perfusion. Third, the indications for PCD is the presence of the vacuum phenomenon in the vertebral space on preoperative CT, which is evidence that the intervertebral disc is degenerated and is the site for bone cement distribution. There is enough room for bone cement during surgery. Fourth, the injection pressure of bone cement in the vertebral space may be high, which need to be injected under x-ray fluoroscopic monitoring. It is very important to prevent bone cement from leaking into the spinal canal at all times.

It can be seen from the above that both treatments could achieve better treatment for for stage III KD with an adjacent disc vacuum sign. At present, IFF surgery was widely recognized (9, 10). However, for some elderly patients who cannot undergo IFF surgery due to complex underlying diseases and great surgical risks, PCD surgery could be considered. The number of patients in the groups were small, this was a single-center study, and the follow-up time needed to be extended to observe the long-term efficacy.

## Conclusion

PCD combined with PVP is an effective treatment for stage III KD with an adjacent disc vacuum sign. Perhaps, the deformity correction and long-term efficacy of PCD combined with PVP are not as good as IFF, but it may be the next best thing to IFF, especially for elderly patients with complex underlying diseases and great surgical risks.

## Data Availability

The raw data supporting the conclusions of this article will be made available by the authors, without undue reservation.

## References

[1] Van Eenenaam DP el-Khoury GY. Delayed post-traumatic vertebral collapse (Kummell’s disease): case report with serial radiographs, computed tomographic scans, and bone scans. Spine. (1993) 18:1236–41. 10.1097/00007632-199307000-00019 8362333

[2] Formica M Basso M Cavagnaro L Formica C Zanirato A Felli L. Kümmell disease: illustrative case for definition criteria. Spine J. (2016) 16:e707–8. 10.1016/j.spinee.2016.03.035 27001113

[3] Ma R Chow R Shen FH. Kummell’s disease: delayed post-traumatic osteonecrosis of the vertebral body. Eur Spine J. (2010) 19:1065–70. 10.1007/s00586-009-1205-4 19949820 PMC2900014

[4] Chen JB Xiao YP Chen D Chang JZ Li T. Clinical observation of two bone cement distribution modes of percutaneous vertebroplasty in the treatment of thoracolumbar Kümmell’s disease. J Orthop Surg Res. (2020) 15:250. 10.1186/s13018-020-01774-8 32646461 PMC7346457

[5] Jiang J Gu FL Li ZW Zhou Y. The clinical efficacy and experience of bipedicular percutaneous vertebroplasty combined with postural reduction in the treatment of Kümmell’s disease. BMC Musculoskelet Disord. (2020) 21:82. 10.1186/s12891-020-3113-z 32033554 PMC7007684

[6] Adamska O Modzelewski K Stolarczyk A Kseniuk J. Is Kummell’s disease a misdiagnosed and/or an underreported complication of osteoporotic vertebral compression fractures? A pattern of the condition and available treatment modalities. J Clin Med. (2021) 10:2584. 10.3390/jcm10122584 34208124 PMC8230888

[7] Yu Y Zeng H Guo E Tang B Fang Y Wu L Efficacy and safety of posterior long-segment fixation versus posterior short-segment fixation for Kummell disease: a meta-analysis. Geriatr Orthop Surg Rehabil. (2022) 13:21514593221107509. 10.1177/21514593221107509 35721367 PMC9203950

[8] Li KC Wong TU Kung FC Li A Hsieh CH. Staging of Kümmell's disease. J Musculoskelet Res. (2004) 8:43–55. 10.1142/S0218957704001181

[9] Patil S Rawall S Singh D Mohan K Nagad P Shial B Surgical patterns in osteoporotic vertebral compression fractures. Eur Spine J. (2013) 22:883–91. 10.1007/s00586-012-2508-4 23053751 PMC3631027

[10] Zhu Y Zhang Z Jiang W Su K Wang Z Wang C Therapeutic efficacy of transpedicular impaction bone grafting with long segmental posterior instrumentation in stage III Kümmell disease. Spine. (2021) 46:907–14. 10.1097/BRS.0000000000003995 34100844

[11] Liu F Chen Z Lou C Yu W Zheng L He D Anterior reconstruction versus posterior osteotomy in treating Kümmell’s disease with neurological deficits: a systematic review. Acta Orthop Traumatol Turc. (2018) 52:283–8. 10.1016/j.aott.2018.05.002 29803679 PMC6146012

[12] Varga PP Jakab G Bors IB Lazary A Szövérfi Z. Experiences with PMMA cement as a stand-alone intervertebral spacer: percutaneous cement discoplasty in the case of vacuum phenomenon within lumbar intervertebral discs. Orthopade. (2015) 44(1):S1–7. 10.1007/s00132-014-3060-1 25875227

[13] Techens C Eltes PE Lazary A Cristofolini L. Critical review of the state-of-the-art on lumbar percutaneous cement discoplasty. Front Surg. (2022) 9:902831. 10.3389/fsurg.2022.902831 35620196 PMC9127498

[14] Jia H Xu B Qi X. Biomechanical evaluation of percutaneous cement discoplasty by finite element analysis. BMC Musculoskelet Disord. (2022) 23:594. 10.1186/s12891-022-05508-1 35725467 PMC9208188

[15] Ortiz AO Bordia R. Injury to the vertebral endplate-disk complex associated with osteoporotic vertebral compression fractures. Am J Neuroradiol. (2011) 32:115–20. 10.3174/ajnr.A2223 20801764 PMC7964944

[16] Shen H Tang W Yin X Shao T Liu X Gu J Comparison between percutaneous short-segment fixation and percutaneous vertebroplasty in treating Kummell’s disease: a minimum 2-year follow-up retrospective study. J Back Musculoskelet Rehabil. (2024) 37:195–203. 10.3233/BMR-230083 37694352 PMC10789354

[17] Wu ZX Gao MX Sang HX Ma ZS Cui G Zhang Y Surgical treatment of osteoporotic thoracolumbar compressive fractures with open vertebral cement augmentation of expandable pedicle screw fixation: a biomechanical study and a 2-year follow-up of 20 patients. J Surg Res. (2012) 173:91–8. 10.1016/j.jss.2010.09.009 21067776

[18] Zhao H Ni CF Huang J Zhao SM Gu WW Jiang H Effects of bone cement on intervertebral disc degeneration. Exp Ther Med. (2014) 7:963–9. 10.3892/etm.2014.1531 24669259 PMC3965156

[19] Jamjoom B Patel S Bommireddy R Klezl Z. Impact of the quantity of intradiscal cement leak on the progression of intervertebral disc degeneration. Ann R Coll Surg Engl. (2017) 99:529–33. 10.1308/rcsann.2017.0083 28853606 PMC5697037

[20] Rose LD Bateman G Ahmed A. Clinical significance of cement leakage in kyphoplasty and vertebroplasty: a systematic review. Eur Spine J. (2024) 33:1484–9. 10.1007/s00586-023-08026-3 37999769

[21] Riesner HJ Kiupel K Lang P Stuby F Friemert B Palm HG. Clinical relevance of cement leakage after radiofrequency kyphoplasty vs. balloon kyphoplasty: a prospective randomised study. Z Orthop Unfall. (2016) 154:370–6. 10.1055/s-0042-104069 27336840

[22] Yamada K Nakamae T Shimbo T Kanazawa T Okuda T Takata H Targeted therapy for low back pain in elderly degenerative lumbar scoliosis: a cohort study. Spine. (2016) 41:872–9. 10.1097/BRS.0000000000001524 26909842

[23] Yamada K Nakamae T Nakanishi K Kamei N Hiramatsu T Okuda T Long-term outcome of targeted therapy for low back pain in elderly degenerative lumbar scoliosis. Eur Spine J. (2021) 30:2020–32. 10.1007/s00586-021-06805-4 33733329

[24] Kiss L Varga PP Szoverfi Z Jakab G Eltes PE Lazary A. Indirect foraminal decompression and improvement in the lumbar alignment after percutaneous cement discoplasty. Eur Spine J. (2019) 28:1441–7. 10.1007/s00586-019-05966-7 31006068

[25] Koch K Szoverfi Z Jakab G Varga PP Hoffer Z Lazary A. Complication pattern after percutaneous cement discoplasty: identification of factors influencing reoperation and length of hospital stay. World Neurosurg. (2023) 178:e700–11. 10.1016/j.wneu.2023.07.148 37544606

[26] Tian QH Lu YY Sun XQ Wang T Wu CG Li MH Feasibility of percutaneous lumbar discectomy combined with percutaneous cementoplasty for symptomatic lumbar disc herniation with Modic type I endplate changes. Pain Physician. (2017) 20:E481–8. 28535556

[27] Ghandour S Pazarlis K Lewin S Isaksson P Försth P Persson C. An ex-vivo model for the biomechanical assessment of cement discoplasty. Front Bioeng Biotechnol. (2022) 10:939717. 10.3389/fbioe.2022.939717 36118564 PMC9478659

[28] Li S Xu B Liu Y Zhang J Xu G Shao P Biomechanical evaluation of spinal column after percutaneous cement discoplasty: a finite element analysis. Orthop Surg. (2022) 14:1853–63. 10.1111/os.13314 35818350 PMC9363717

[29] Farshad M Götschi T Bauer DE Böni T Laux CJ Kabelitz M. Long-term outcome of patients with adolescent idiopathic scoliosis seeking nonoperative treatment after a mean follow-up of 42 years. Spine Deform. (2022) 10:1331–8. 10.1007/s43390-022-00541-5 35819723 PMC9579110

